# Serum levels of *N*-acetyl-aspartate in migraine and tension-type headache

**DOI:** 10.1007/s10194-012-0448-3

**Published:** 2012-04-17

**Authors:** Marina de Tommaso, Edmondo Ceci, Carmela Pica, Maria Trojano, Marianna Delussi, Giovanni Franco, Paolo Livrea, Maddalena Ruggieri

**Affiliations:** 1 Neuroscience and Sensory System Department, Bari Aldo Moro University, Policlinico General Hospital, Neurological Building, Piazza Giulio Cesare 11, 70124 Bari, Italy; 2 Department of Animal Production, Faculty of Veterinary Medicine of Bari-Valenzano, Bari, Italy

**Keywords:** Serum *N*-acetyl-aspartate, Migraine, Tension-type headache

## Abstract

Serum levels of *N*-acetyl-aspartate (NAA) may be considered a useful marker of neuronal functioning. We aimed to measure serum NAA in cohorts of migraine and tension-type headache patients versus controls, performing correlations with main clinical features. A total of 147 migraine patients (including migraine without aura, with aura and chronic migraine), 65 tension-type headache (including chronic and frequent episodic tension-type headache) and 34 sex- and age-matched controls were selected. Serum was stored at −80 °C. Quantification of NAA was achieved by the standard addition approach and analysis was performed with liquid-chromatography–mass-spectrometry (LC/MS) technique. The NAA levels were significantly decreased in migraine group (0.065 ± 0.019 mol/L), compared with both tension-type headache patients (0.078 ± 0.016 mol/L) and controls (0.085 ± 0.013 mol/L). Control subjects were significantly different from migraine with and without aura and chronic migraine, who differed significantly from episodic and chronic tension-type headache. Migraine with aura patients showed lower NAA levels when compared to all the other headache subtypes, including migraine without aura and chronic migraine. In the migraine group, no significant correlation was found between NAA serum levels, and headache frequency, allodynia and interval from the last and the next attack. The low NAA in the serum may be a sign of neuronal dysfunction predisposing to migraine, probably based on reduced mitochondria function.

## Introduction

Migraine is an invalidating disorder of neurovascular origin, which causes remain partly unknown. Altered brain excitability has been supposed, based on genetic mutations and/or polymorphisms of chromosomes, yet to be determined, which regulate the metabolism of neuronal mitochondrial energy, neurotransmitters and ion canals of the CNS [1]. Previous studies performed by proton magnetic resonance spectroscopy (1H-MRS), a non-invasive technique able to evaluate cerebral metabolites, found a consistent decrease of the *N*-acetylaspartate (NAA) signal in the brain of migraine with aura patients during the intermittent photic stimulation [2]. Neurons are the only source of *N*-acetyl-aspartate (NAA). This molecule, which assesses neuronal integrity, leaves central nervous system (CNS) through astrocytes and then it is reversed into circulation and excreted by kidney in urine samples [3–5]. The NAA is considered, in particular, a marker of axonal integrity. It is synthesized and located prevalently in neural mitochondria [3–5], and in fact it has been taken as a marker of mitochondrial functioning [6]. In the study by Sarchielli et al. [2], authors postulated that the greater decrease in NAA at baseline (before visual stimulation) suggested a less efficient mitochondrial functioning in migraine with aura compared with patients without aura, who, on the other hand, showed a slight but not significant NAA decrease during photic stimulation compared with controls. In both migraine patients and controls, the NAA reduction was completely reversible, and linked with the metabolic shift characterizing the alteration in cortical homeostatic mechanisms induced by intermittent photic stimulation. Another study about metabolite concentration in the thalamus of migraine and trigeminal neuralgia patients, performed by 1H-MRS, showed a decrease of NAA/choline (Cho) ratio in the thalamus of migraine patients [7]. A reduction in the NAA/creatine ratio was also found at the thalamic level in patients affected by trigeminal neuropathic pain when compared with other non-neuropathic trigeminal painful syndromes, as a sign of the structural modification occurring in the course of central sensitization processing [8].

Recent studies outlined the validity of NAA measurements in biological fluids, serum and cerebrospinal, using a gas-chromatography–mass-spectrometry (GC–MS) method in patients with neurological diseases [9–11]. Our group found that serum NAA, measured by liquid-chromatography–MS (LC–MS), was significantly reduced in 141 healthy controls with advancing age, probably due to physiological neuronal loss and exhausted mechanisms of neurodegeneration [12], while high levels of serum NAA were found in different conditions of neurodegeneration [13–15]. The first route for NAA clearance is its transfer from neurons to oligodendrocytes, where the enzyme Asparto-Acylase cleaves the acetate moiety for use in fatty acid and steroid synthesis. In pathological conditions as amyotrophic lateral sclerosis or Huntington’s disease, the neuronal damage may produce a continuous efflux of NAA to extracellular space. Hence, the enhanced NAA is preferentially taken up into astrocytes and finally excreted to the circulation. The reduced NAA observed by 1H-MRS in these conditions may suggest that its increase into circulation may be due to its progressive intra-cellular reduction and extracellular wash out [13, 14].

On this basis, serum levels of NAA may be considered a useful marker of neuronal functioning. In migraine patients, the reduction of NAA seen by 1H-MRI was attributed to an energetic problem linked to mitochondria [2]. In this hypothesis the reduced NAA production into the brain should also cause changes of its level in CSF and circulation. We aimed to measure serum NAA in cohorts of migraine and tension-type headache patients versus controls, and to perform clinical correlations, in order to test the hypothesis that possible modification of NAA metabolism in the brain may be specific for migraine or linked with central processing of pain, occurring in both types of primary headaches.

## Methods

Consecutive out-patients, from 1 January 2010 to 1 June 2010, who came to the Headache Ambulatory of the Neurophysiopathology of Pain Unit of Neuroscience and Sensory System Department of Bari University were enrolled. The inclusion criteria were a diagnosis of primary headache, migraine or tension-type headache, according to International Classification of Headache Disorders, 2nd edn (ICHD-II) criteria [16], specifically migraine without aura (cod. 1.1), migraine with aura (cod. 1.2), chronic migraine (cod. 1.5.1), episodic frequent tension-type headache (cod. 2.2), chronic tension-type headache (cod. 2.3). We did not include medication overuse headache (cod. 8.2) for the possible interference of overused drug with NAA serum levels. Exclusion criteria for patients and controls were the presence of general medical or neurological and psychiatric disorders, according to DSMIV-R, analgesic or symptomatic drugs taking in the last 24 h, CNS-acting drug therapies in the last 2 months. Basing on these criteria, we were able to select a total of 212 patients and 34 age- and sex-matched controls, as detailed in Table 1. All patients were submitted to the clinical evaluation, according to de Tommaso et al. [17]. The frequency of headache, as the average number of days with headache/month, computed across 3 months, the Migraine Disability Assessment scale-MIDAS score, in the Italian version [18, 19], the allodynia questionnaire [20], and the Short-Form 36 (SF-36) Health Survey [21] were also considered to perform a correlation with NAA serum levels found in headache groups. In addition, we checked the interval from the last and the next headache episode, the latter during the clinical follow-up or by telephonic interview.

**Table 1:** Age and sex of the considered headache patients and controls

Main ICHD II group	Headache type	No.	Sex	Age
Migraine Cod. 1	Migraine without aura cod 1.1	90	F 68 M 31	Mean 37.66 SD 12.84 CI 35.10–40.22
	Migraine with aura cod. 1.2	16	F 10 M 6	Mean 33 SD 9.74 CI 24.98–43
	Chronic migraine cod. 1.5.1	35	F 28 M 7	Mean 46.62 SD 10.37 CI 42.3–50.95
Tension-type headache Cod. 2	Chronic tension-type headache cod. 2.3	43	F 27 M 16	Mean 42.76 SD 15.83 CI 33.2–52.33
	Episodic frequent tension-type headache cod 2.2	22	F 12 M 10	Mean 36 SD 4.93 CI 33.4–39.58
Controls		34	F 23 M 11	Mean 41.47 SD 10.37 CI 27.85–45.08

The Chi-square for sex was not significant, considering both headache groups and types, as well as the ANOVA test with age as variable: for headache group *F*: 1.21 DF 2 n.s.; for headache type; *F*: 1.98 DF 5 n.s.

The time of the day when the blood sample was taken was in the morning at 8 a.m. The sample was taken from the anticubital vein and the patients were fasting.

Serum was stored at −80 °C. Quantification of NAA was achieved by the standard addition approach and analysis was performed with LC/MS technique [12].

A linear regression fitting (*y* = *ax* + *b*) was done on all the calibration data. The calculations of the detection limits for the compound studied were based on a signal-to-noise ratio of 3. The limit of detection was 0.1 μmol/L. The instrumental precision was calculated by considering the repeatability of four measurements of chromatographic peak areas at two amount levels. The imprecision of the whole procedure (pre-treatment and quantification) expressed as coefficient of variation (CV) was assessed using pooled serum samples that were repeatedly analyzed by standard addition. The intraday (*n* = 10 times) and interday (*n* = 10 times) CV were, respectively, 2.1 and 6.2 % [12]. The recovery calculations for serum samples showed a mean of 87 %. Accuracy was evaluated by comparing the results obtained in this study with those measured independently by GC–MS in a reference laboratory. Data were acquired by Thermo Electron Corporation Excalibur software [12]. Readers were blinded to all clinical informations.

The local Ethic Committee of Bari Policlinico General Hospital approved the study and all patients and controls gave their informed consent.

## Statistical analysis

The NAA serum levels were first compared across the groups considered in Table 1, performing a one-way ANOVA test with a post hoc Bonferroni test. The same test was also applied across the main headache types (Table 1). In the headache groups, the NAA serum levels were correlated with the clinical features, by the means of Spearman correlation test. The SPSS software, ver. 19 was utilized.

## Results

**Fig. 1:**
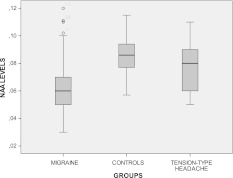
Serum levels of *N*-acetyl-aspartate (NAA) in the main primary headache groups and controls

**Fig. 2:**
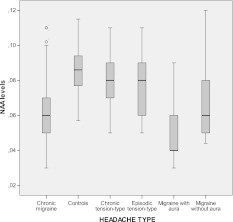
Serum levels of *N*-acetyl-aspartate (NAA) in the main primary headache types and controls

The NAA levels were significantly decreased in migraine group (0.065 ± 0.019 mol/L), compared with both tension-type headache patients (0.078 ± 0.016 mol/L) and controls (0.085 ± 0.013 mol/L) (ANOVA: *F*: 19.25, DF 2, *p* = 0.0001. Bonferroni test: migraine vs. controls *p* < 0.0001; migraine vs. tension-type headache *p* = 0.002; tension-type headache vs. controls n.s.) (Fig. 1). Considering the headache types, the ANOVA test was also significant (*F* = 10.32, DF 5, *p* < 0.0001). The Bonferroni test indicated that control subjects were significantly different from all the migraine types (*p* < 0.0001), and migraine with and without aura and chronic migraine differed significantly from episodic and chronic tension-type headache (*p* < 0.05). However, migraine with aura patients showed lower NAA levels when compared to all the other headache subtypes, including migraine without aura (*p* < 0.01) and chronic migraine (*p* < 0.05) (Fig. 2). In the migraine group, no significant correlation was found between NAA serum levels, and headache frequency, duration, MIDAS and allodynia and SF-36 scores. The interval from the last headache episode was on average 52.4 ± 12 h in migraine group and 58 ± 11.2 in tension-type headache, while headache followed NAA evaluation 45.5 ± 20.5 h in migraine group, and 48.5 ± 18.4 h in tension-type headache. Giving that one of the exclusion criteria was the recent symptomatic treatment, only one migraine without aura patient, two chronic migraine and three chronic tension-type headache were submitted to NAA serum evaluation during the course of headache. However, we found no correlation between serum NAA and the interval from the last and the next attack.

## Discussion

In this study, serum NAA levels in migraine patients were significantly lower than in tension-type headache and healthy controls. This result seems to confirm previous findings by Sarchielli et al. [2], who described reduced brain NAA signal in migraine with aura examining 1H-magnetic resonance spectroscopy (MRS) during cortical activation induced by photic stimulation. The provisional link between our results and those provided by MRS are still speculative, though LC–MS seems an available method to dose this marker of neuronal functioning in the serum. Many studies have focalized attention on NAA serum or cerebrospinal fluid (CSF) levels in different neurological conditions, with or without MRS evaluations; for example, a decrease of NAA in the motor cortex in amyotrophic lateral sclerosis (ALS) patients was reported by 1H-MRS investigations [22].

Although our data are preliminary, possible mechanisms underlying the observed decrease of serum NAA have to be considered. The first route for NAA clearance is its transfer from neurons to oligodendrocytes, where the enzyme Asparto-Acylase cleaves the acetate moiety for use in fatty acid and steroid synthesis. In pathological conditions as ALS, the damage of motor neurons may produce a continuous efflux of NAA to extracellular space. Hence, the enhanced NAA is preferentially taken up into astrocytes and finally excreted to the circulation [23], whereas in other diseases, such as in acute phase of ischemic stroke, a more significant increase of NAA levels was reported, in particular, in the first 2 days after ischemic damage [9]. Similar results have been found in serum samples of multiple sclerosis (MS) patients [15]. These data underlined that inflammatory or ischemic acute events are all characterized by a major increase of NAA leakage following acute neuronal damage, whereas in neurodegenerative processes the NAA increase is less, following a slower but progressive pathological neuronal impairment.

In agreement with in vivo previous 1H-MRS studies [24], we found an age-related decline of NAA serum levels in healthy subjects [12]. This suggests that in a physiological event, as aging, the reduced serum NAA might be a result of a reduced NAA synthesis, secondary to age-related neuronal energetic impairment [25]. In our migraine population the low NAA serum levels were independent of possible confounding factors such as age and sex. This decrease could therefore be a consequence of an energetic problem linked to mitochondria, perhaps based on an enzyme deficit, so the molecule is unable to exit from mitochondria to CSF. We were unable to detect NAA CSF levels for ethical problem, so we can only postulate its lower levels in CSF too.

We can speculate that a biomarker in serum cannot actually localize the specific affected brain region, but it generally reflects a damage, perhaps to neuronal mitochondria. Our findings may presently indicate that NAA in serum may be a sign of neuronal dysfunction predisposing to migraine, probably based on reduced mitochondria function.

In addition, the NAA reduction detected by 1H-MRS was specially observed in migraine with aura patients under the cortical activation induced by photic stimulation, while the NAA reduction observed in migraine without aura was only slightly more evident than in controls [2]. In our migraine series, the NAA levels were reduced in both types of migraine, including the chronic form evolved from migraine without aura, though patients experiencing aura symptoms were characterized by the lowest serum NAA. The NAA reduction in the circulation is a quite unspecific finding; however, following the reason that it may be a sign of reduced neuronal [5] and glial [26] mitochondria function, the hypothesis of a deficient energy metabolism may be supposed for both types of migraine [27]. Mitochondrial dysfunction has been identified as one potential cause of epileptic seizures and altered brain excitability [28], and in this sense the measure of serum NAA in epileptic patients and more specifically in MELAS and MERFF syndromes would confirm the origin of the low NAA levels found in migraine. Moreover, cerebral mitochondrial injury seems to be related to cortical spreading depression [29], so assuming that this neuronal phenomenon may precede any form of migraine, independently from the perception of aura symptoms [30], the NAA reduction may be the consequence of the migraine episodes repetition. However, in contrast with this hypothesis is the lack of correlation between NAA levels, and frequency of migraine, disease duration and time from the last attack. Presently, the link between low NAA in the serum and cortical spreading depression phenomenon may not be supported by present data, which only indicate low serum NAA as a sign of neuronal dysfunction predisposing to migraine, with a more evident phenotypic expression in patients experiencing aura symptoms. Migraine and tension-type headache patients, who in clinical practice may exhibit similar associated features [31], seem different in regard to this marker of neuronal function. In fact, the NAA levels were within the normal ranges even in those patients experiencing frequent tension-type headache episodes, suggesting that the neuronal structural modification occurring in the course of central sensitization processing [8] would not be a relevant phenomenon for NAA serum reduction. This may also be further confirmed by the lack of correlation between serum NAA and allodynia symptoms detected in migraine group. These results could not reduce the validity of the theory about the central origin of pain in tension-type headache [32], rather they would indicate that low serum NAA is not related to central mechanisms of pain processing, but to neuronal abnormalities which may specifically predispose to migraine.

Though our study may contribute to outline the different pathophysiological basis of migraine and tension-type headache and to suggest the similarity between migraine with and without aura, it has to be considered preliminary, in view of the small series and the lack of precise understanding of brain conditions underlying changes in serum NAA. This may be considered a sign of neuronal dysfunction predisposing to migraine, probably based on reduced mitochondria function. However, the results of this study should be considered preliminary and have to be confirmed by a prospective study with 1H-MRS spectroscopy and the contemporary measurement of NAA peripheral levels.
